# Alpha7 nicotinic acetylcholine receptor activation attenuates allergic airway inflammation and is associated with heme oxygenase-1 induction

**DOI:** 10.3389/fimmu.2026.1808271

**Published:** 2026-04-23

**Authors:** Jun Iriki, Susumu Fukahori, Yasushi Obase, Yasuna Nakamura, Ryusuke Umene, Chia-Hsien Wu, Yuri Yamada, Shinnosuke Takemoto, Takahiro Takazono, Noriho Sakamoto, Chizu Fukushima, Tomoya Nishino, Hiroshi Mukae, Tsuyoshi Inoue

**Affiliations:** 1 Department of Respiratory Medicine, Nagasaki University Graduate School of Biomedical Sciences, Nagasaki, Japan; 2 Department of Respiratory Medicine, Nagasaki University Hospital, Nagasaki, Japan; 3 Department of Physiology of Visceral Function and Body Fluid, Graduate School of Biomedical Sciences, Nagasaki University, Nagasaki, Japan; 4 Department of Nephrology, Nagasaki University Graduate School of Biomedical Sciences, Nagasaki, Japan; 5 Department of Anesthesiology and Intensive Care Medicine, Nagasaki University Graduate School of Biomedical Sciences, Nagasaki, Japan; 6 Department of Infectious Diseases, Nagasaki University Graduate School of Biomedical Sciences, Nagasaki, Japan; 7 Clinical Research Center, Nagasaki University Hospital, Nagasaki, Japan

**Keywords:** airway epithelial cell, allergic airway inflammation, heme oxygenase-1, thymic stromal lymphopoietin, α7nAChR

## Abstract

**Background:**

Activation of the α7 nicotinic acetylcholine receptor (α7nAChR) exerts anti-inflammatory effects; however, its role in allergic airway inflammation remains poorly understood. This study investigated how GTS-21 treatment, consistent with α7nAChR activation, influences airway inflammation and epithelial cytokine responses.

**Methods:**

A murine model of allergic airway inflammation was established in female C57BL/6J mice by repeated house dust mite (HDM) exposure. Mice received intratracheal GTS-21 or phosphate-buffered saline (PBS) prior to each HDM challenge. Airway inflammation and cytokine levels were then evaluated. *In vitro*, BEAS-2B human airway epithelial cells were treated with GTS-21 or PBS, followed by stimulation with HDM and lipopolysaccharide (LPS). Thymic stromal lymphopoietin (TSLP) production was quantified, and RNA sequencing was performed to analyze gene expression changes.

**Results:**

Intratracheal GTS-21 administration significantly attenuated HDM-induced eosinophilic airway inflammation in mice and reduced TSLP and type 2 cytokine levels. In BEAS-2B cells, GTS-21 treatment similarly suppressed LPS- and HDM-induced TSLP production. RNA sequencing revealed that GTS-21 treatment upregulated heme oxygenase-1 (HO-1) expression. Increased HO-1 expression was associated with reduced TSLP production under these experimental conditions.

**Conclusion:**

GTS-21 attenuated allergic airway inflammation and suppressed epithelial TSLP production in association with increased HO-1 expression. These findings suggest that cholinergic receptor-associated signaling may modulate epithelial inflammatory responses in allergic airway inflammation.

## 1 Introduction

Asthma, characterized by variable airflow limitation, airway hyperresponsiveness, and persistent inflammation, is a chronic inflammatory disorder of the airways (1). Although the combination of inhaled corticosteroids (ICS) and long-acting β2-agonists is the primary treatment strategy and is effective in most patients, approximately 10% of patients experience severe asthma that remains poorly controlled despite high-dose ICS (2). Consequently, additional therapies, including long-acting muscarinic antagonists, leukotriene modifiers, or biologics, are often required for these patients, which increase the risk of adverse effects and contribute to a significant economic burden (3). Hence, developing novel anti-inflammatory strategies, particularly for severe and steroid-resistant asthma, is crucial.

Recent advances in the study of neuroimmune interactions have highlighted the role of the cholinergic anti-inflammatory pathway (CAP). This reflex-mediated pathway suppresses cytokine production through efferent vagus nerve activity, which activates alpha7 nicotinic acetylcholine receptors (α7nAChRs) on immune cells (4). Notably, vagus nerve stimulation (VNS) reduced inflammation in experimental models of endotoxemia (5), inflammatory bowel disease (6), rheumatoid arthritis (7), and kidney ischemia–reperfusion injury (8). Noninvasive VNS may improve lung function and alleviate dyspnea in patients with respiratory disorders, including asthma (9).

Within the pulmonary system, α7nAChRs are expressed on immune cells (e.g., alveolar macrophages and group 2 innate lymphoid cells (ILC2s) (10)) and structural cells (e.g., airway epithelial cells (11)). Activation of α7nAChRs inhibits ILC2 activation and reduces airway inflammation in allergic models (12). However, the precise mechanisms through which α7nAChR agonists exert their anti-inflammatory effects remain unclear.

Thymic stromal lymphopoietin (TSLP), an epithelial cell-derived cytokine, is a key upstream regulator of type 2 inflammation (13). TSLP promotes dendritic cell-mediated Th2 differentiation and activates lung-resident ILC2s while also contributing to steroid resistance in these cells. In asthma, various environmental stimuli, such as allergens, pathogens, and pollutants, trigger TSLP production (14, 15). Notably, in murine models of atopic dermatitis, α7nAChR activation in keratinocytes suppresses TSLP production (16).

**Figure 1:**
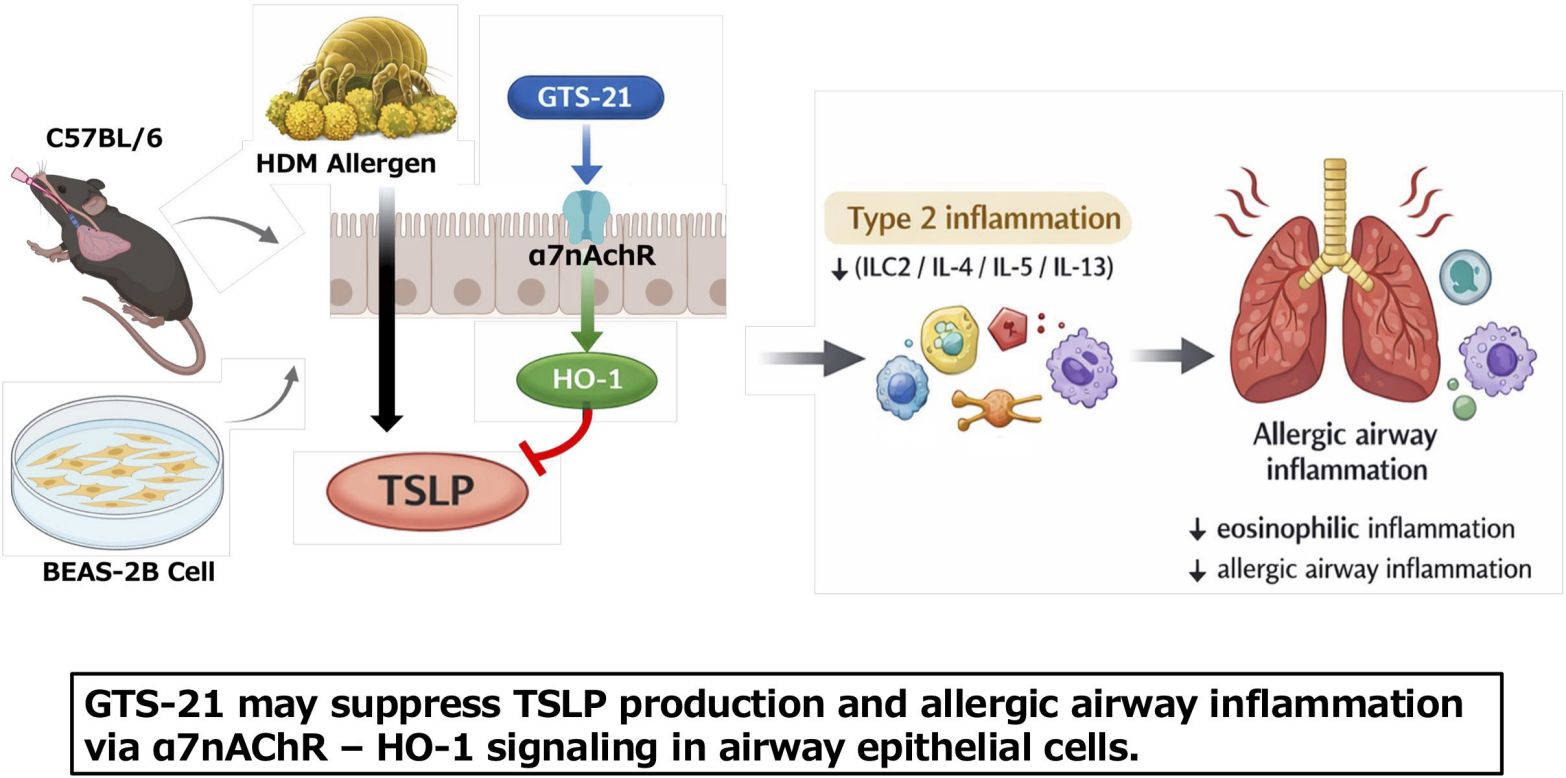
Graphical abstract of the study. The α7 nicotinic acetylcholine receptor (α7nAChR) partial agonist GTS-21 attenuated allergic airway inflammation and suppressed epithelial TSLP production in association with increased HO-1 expression. In a murine model of HDM-induced allergic airway inflammation (C57BL/6) and in BEAS-2B airway epithelial cells, activation of α7nAChR by GTS-21 was associated with induction of heme oxygenase-1 (HO-1) and reduced production of thymic stromal lymphopoietin (TSLP). Reduced TSLP levels may subsequently limit type 2 inflammatory responses, thereby contributing to attenuation of eosinophilic airway inflammation.

Based on this background, we hypothesized that GTS-21 treatment, consistent with α7nAChR activation, may modulate airway epithelial inflammatory responses and suppress TSLP production, thereby contributing to attenuation of allergic airway inflammation. We therefore investigated the effects of GTS-21 in a murine model of HDM-induced allergic airway inflammation and in BEAS-2B airway epithelial cells, and explored associated molecular changes using RNA sequencing. A graphical abstract summarizing the study is shown in **Figure 1**.

## 2 Methods

### 2.1 Mice

Female C57BL/6J mice (6–8 weeks old) were obtained from the Central Laboratory for Experimental Animals (CLEA Japan, Tokyo, Japan) and acclimated in the animal facility for 1 week before the experiments. Mice were housed in standard cages under a 12-h light/dark cycle with ad libitum access to food and water. All animal experiments were approved by the Institutional Animal Care and Use Committee of Nagasaki University (approval no. 2206231799) and were conducted in accordance with institutional guidelines and the relevant Japanese regulations for animal experimentation. All efforts were made to minimize animal suffering.

### 2.2 HDM-induced allergic airway inflammation model in mice

**Figure 2:**
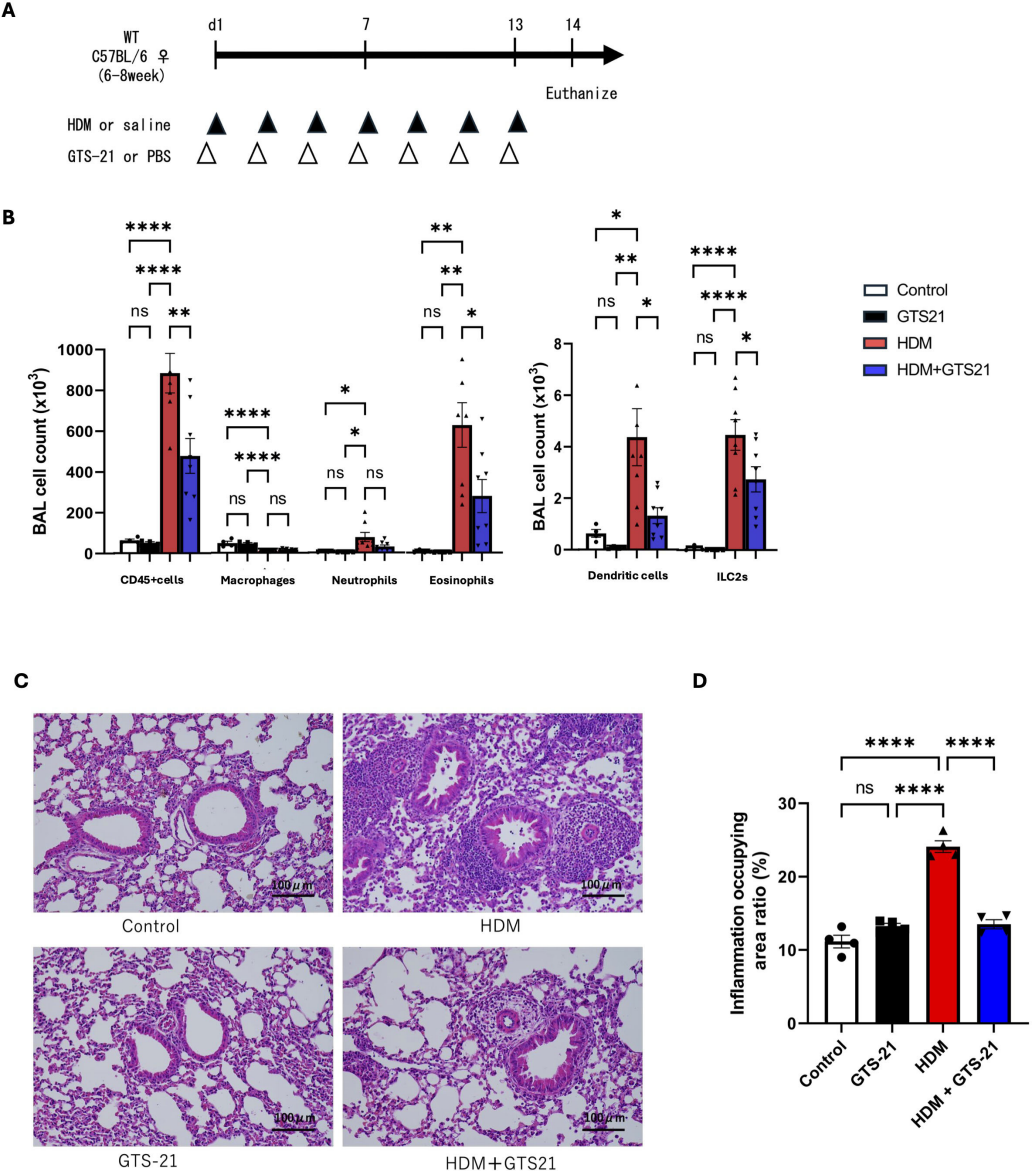
GTS-21 suppresses allergic airway inflammation in a murine model of HDM-induced allergic airway inflammation **(A)** Schematic of the experimental protocol. Mice received intratracheal administrations of HDM extract (30 μg) every other day for a total of seven doses. GTS-21 (125 μg) was administered intratracheally 30 min before each HDM challenge. Mice were euthanized 24 h after the final administration. **(B)** Immune cell populations in BALF were quantified via flow cytometry. **(C)** HE staining of lung tissue sections. Scale bar = 100 μm; n = 4 mice. **(D)** Lung inflammation was quantified as the percentage of the inflamed area with ImageJ software based on HE-stained sections. Points represent individual mice **(B, D)**, and bars represent the mean ± standard error of the mean **(B, D)** and are representative of two independent experiments. n = 4–8 mice per group. Statistical analysis was performed using two-way analysis of variance followed by Tukey’s *post hoc* test. n.s., not significant. ∗P < 0.05, ∗∗P < 0.01, ∗∗∗P < 0.001, ∗∗∗∗P < 0.0001. HDM, house dust mite; BALF, bronchoalveolar lavage fluid; HE, hematoxylin and eosin.

A murine model of HDM-induced allergic airway inflammation was established using HDM (Dermatophagoides farinae crude extract; LSL, Tokyo, Japan) as previously described (17, 18). C57BL/6J wild-type (WT) mice were anesthetized via intraperitoneal injection of a mixture containing medetomidine (0.3 mg/kg), butorphanol (5 mg/kg), and midazolam (4 mg/kg). Mice then received intratracheal instillation of 30 µg of HDM or normal saline (Otsuka, Tokushima, Japan) every other day for a total of seven administrations. Additionally, 125 µg of GTS-21 (SML0326, Sigma-Aldrich, St. Louis, MO, USA) or phosphate-buffered saline (PBS) was administered intratracheally 30 min prior to each HDM or saline challenge. Intratracheal administration was performed under anesthesia by non-surgical intratracheal instillation and was not performed intranasally. Mice were euthanized 24 h after the final injection for downstream analyses (**Figure 2A**).

### 2.3 Flow cytometry

Bronchoalveolar lavage fluid (BALF) was collected by instilling the lungs twice with 1 mL of sterile normal saline under anesthesia. The lavage fluid was centrifuged at 500 × *g* for 5 min at 4 °C to obtain a single-cell suspension.

To block Fc receptors, cells were incubated with anti-CD16/32 antibodies (Biolegend, San Diego, CA, USA). Surface staining was performed by incubating the cells for 30 min at 4 °C with the following antibodies: CD45.2 APC (eBioscience, San Diego, CA, USA), CD11b FITC (eBioscience), CD11c PECy7 (eBioscience), Ly6G eFluor 450 (eBioscience), MHC class II eFluor 506 (eBioscience), and Siglec-F Super Bright 600 (eBioscience) to identify dendritic cells, neutrophils, and eosinophils (19). To identify ILC2s, cells were stained with Lineage FITC (Biolegend), CD90.2 BV421 (Biolegend), and IL-33R APC-eFluor 780 (eBioscience) (20, 21). After staining, cells were washed twice with 1× fluorescence-activated cell sorting buffer (BD Biosciences, Franklin Lakes, NJ, USA) and incubated with 1 µg/mL 7-AAD (Thermo Fisher Scientific, Waltham, MA, USA) in the same buffer for 10 min at 4 °C to exclude dead cells. Flow cytometry data were analyzed using FlowJo software (v10.10.0; BD Biosciences).

The flow-cytometry gating strategy is provided in the **Supplementary Material**.

### 2.4 Lung histology and immunohistochemistry

Lung tissues were fixed in 4% formaldehyde after sampling and embedded in paraffin for histological and immunohistochemical analysis. Lung sections (5 μm thick) were stained with hematoxylin and eosin for histological evaluation.

For immunohistochemical staining of TSLP, HO-1, and α7nAChR, sections were deparaffinized and subjected to antigen retrieval using 10 mM sodium citrate buffer (pH 6.0; RM102-C, LSI Mediante, Tokyo, Japan) at 120 °C for 10 min. Endogenous peroxidase activity was quenched by treating the sections with 3% hydrogen peroxide in distilled water for 15 min. Non-specific binding was blocked with 10% normal goat serum (06349-64; Nacalai Tesque, Kyoto, Japan) for 1 h at room temperature. Sections were then incubated overnight at 4 °C with either rabbit anti-TSLP antibody (1:200; ab115700, Abcam, Cambridge, UK), rabbit anti-heme oxygenase-1 (HO-1) antibody (1:200; SPC-112, StressMarq, Victoria, BC, Canada), or rabbit anti-α7nAChR antibody (1:200; ab216485, Abcam) diluted in the blocking solution. After washing, the sections were incubated with horseradish peroxidase (HRP)-conjugated goat anti-rabbit immunoglobulin antibody (414341, Nichirei, Saitama, Japan) for 1 h at room temperature. Positive staining was visualized using 3,3′-diaminobenzidine tetrahydrochloride (K3468, Dako, Santa Clara, CA, USA). Finally, sections were counterstained with Mayer’s hematoxylin, dehydrated, and mounted. Negative controls were included for all specimens.

### 2.5 Histological analysis

Stained lung sections were photographed and analyzed using ImageJ software (National Institutes of Health, Bethesda, MD, USA). The degree of inflammatory cell infiltration, the area of TSLP immunostaining and HO-1 intensity were quantified.

For each sample, three bronchi were randomly selected at 200× magnification using a light microscope (Digital Sight 10, Nikon, Tokyo, Japan). Inflammatory changes and TSLP-positive areas were quantified as a percentage using ImageJ. Airway epithelial regions were manually selected as regions of interest (ROIs), and the mean HO-1 intensity within the airway epithelium was measured for each airway and averaged per sample. Results are expressed as the mean value per sample. Airway-focused quantification was interpreted cautiously because TSLP staining in lung tissue cannot be assumed to originate exclusively from epithelial cells.

### 2.6 BEAS-2B cell culture

Immortalized human lung bronchial epithelial BEAS-2B cells were cultured in Roswell Park Memorial Institute-1640 medium (R8758, Sigma-Aldrich) supplemented with 10% (v/v) fetal bovine serum (F7524, Lot #BCBT 3830, Sigma-Aldrich), 100 units/mL penicillin G sodium, and 100 μg/mL streptomycin sulfate (15140122, Thermo Fisher Scientific). Cultures were maintained at 37 °C in a humidified incubator with 5% CO_2_ and 95% air. Cells at passages 10–20 were used for all experiments. For stimulation assays, 1.4 × 10^5^ cells were seeded in 12-well plates and incubated for 48 h.

GTS-21 (100 μM; SML0326, Sigma-Aldrich), an α7nAChR partial agonist, was added 30 min before stimulation with 50 or 100 μg/mL HDM and 1 or 10 μg/mL lipopolysaccharide (LPS; 4391, Sigma-Aldrich). Cells were then incubated for an additional 6 h. Human HO-1 protein (1 ng/mL; 3776-HM, R&D Systems, Minneapolis, MN, USA) was added 30 min before stimulation, similar to GTS-21.

### 2.7 RNA extraction and quantitative real-time PCR

Total RNA was extracted using the FastGene RNA Basic Kit (FG-80006; NIPPON Genetics, Tokyo, Japan). RNA concentration and purity were assessed via spectrophotometric determination of the 260/280 absorbance ratio using a Synergy LX plate reader (BioTek Instruments, Winooski, VT, USA). Complementary DNA was synthesized from the extracted RNA using the PrimeScript RT Master Mix (RR036A; Takara Bio, Shiga, Japan) following the manufacturer’s instructions. qPCR was performed using iTAC Universal SYBR Green Supermix (1725121, Bio-Rad, Tokyo, Japan) to measure relative mRNA expression of target genes. Glyceraldehyde-3-phosphate dehydrogenase (*GAPDH*) was used as an internal control. Amplification was performed using the CFX Connect Real-Time PCR Detection System (Bio-Rad), and relative gene expression was analyzed using the ΔΔCt method. The primer sequences were as follows: *GAPDH* forward, 5′-CCTCAACGACCACTTTGTCA -3′, reverse, 5′-TTACTCCTTGGAGGCCATGT-3′; HO-1 forward, 5′-CCAGGCAGAGAATGCTGAGTTC-3′, reverse, 5′-AAGACTGGGCTCTCCTTGTTGC-3′.

### 2.8 RNA-seq

RNA-seq was performed by Novogene Co. (Beijing, China) using RNA extracted from BEAS-2B cells that were incubated with LPS and HDM extract for 6 h following treatment with either GTS-21 or PBS. Differential gene expression between the two groups was analyzed through pairwise comparison, and enrichment analysis was conducted using RNAseqChef (https://imeg-ku.shinyapps.io/RNAseqChef/) as described previously (22).

### 2.9 Western blotting

BEAS-2B cells were lysed in ice-cold radioimmunoprecipitation assay buffer (FUJIFILM Wako, Osaka, Japan) supplemented with a protease inhibitor (cOmplete Mini, Roche, Basel, Switzerland). Protein concentration was determined using a bicinchoninic acid protein assay kit (Pierce Biotechnology, Waltham, MA, USA). Protein samples were denatured by heating at 95 °C for 10 min in LDS sample buffer and reducing agent (Thermo Fisher Scientific). Proteins were separated via sodium dodecyl sulfate–polyacrylamide gel electrophoresis using MOPS SDS running buffer (Thermo Fisher Scientific) and transferred onto a polyvinylidene fluoride membrane (Millipore, Burlington, MA, USA). Membranes were blocked for 1 h at 4 °C with 5% skim milk (FUJIFILM Wako) in Tris-buffered saline with 0.1% Tween-20. Subsequently, membranes were incubated overnight at 4 °C with primary antibodies against HO-1 (StressMarq) and β-actin (Cell Signaling Technology, Danvers, MA, USA). After washing, membranes were incubated with HRP-conjugated goat anti-rabbit IgG secondary antibody (Thermo Fisher Scientific) for 1 h at room temperature. Protein bands were visualized using enhanced Chemiluminescence Plus Western Blotting Substrate (Pierce Biotechnology). β-actin was used as a loading control for normalization.

### 2.10 Enzyme-linked immunosorbent assay

Human TSLP, interleukin (IL)-33, and IL-25 levels in cell culture supernatants were measured using ELISA kits (R&D Systems). Similarly, mouse TSLP, IL-33, IL-25, IL-4, IL-5, and IL-13 levels in lung homogenate supernatants were quantified using corresponding ELISA kits from the same manufacturer. Absorbance was measured using a Synergy LX plate reader (BioTek Instruments).

### 2.11 Statistical analysis

All statistical analyses were performed using GraphPad Prism 9 (GraphPad Software, San Diego, CA, USA). Data are presented as mean ± standard error of the mean (SEM). Formal testing for normality was not performed in the original analysis. Comparisons between two groups were performed using Student’s t-test. Comparisons among multiple groups were performed using one-way or two-way analysis of variance, followed by appropriate *post hoc* multiple-comparison testing as indicated in the figure legends. P < 0.05 was considered statistically significant. These experiments were exploratory in nature, and no formal *a priori* power calculation was performed.

Mice were allocated to treatment groups in a randomized manner. Histological image analysis was performed in a blinded fashion. Flow-cytometric and ELISA analyses were conducted using predefined analysis criteria.

## 3 Results

### 3.1 GTS-21 suppresses allergic airway inflammation in a murine model of HDM-induced allergic airway inflammation

To examine the effects of a cholinergic receptor-targeting agent on allergic airway inflammation, we established a murine model of HDM-induced allergic airway inflammation following HDM exposure (**Figure 2A**). In this model, mice received intratracheal administration of GTS-21, an α7nAChR partial agonist, or PBS 30 min before each HDM challenge. A total of seven administrations were performed every other day, and mice were euthanized 24 h after the final exposure. Flow cytometric analysis of BALF demonstrated significantly lower numbers of CD45+ leukocytes, CD11c^−^ Siglec-F+ eosinophils, CD11c+ MHC class II+ dendritic cells, and Lineage^−^ CD90.2+ ST2+ ILC2s in the GTS-21-treated group than in the PBS-treated group (**Figure 2B**).

Subsequently, histological analysis was performed using lung sections from mice treated as described above. Inflammatory cell infiltration—predominantly eosinophils—around the airways and blood vessels was greater in the HDM-challenged group than in the control group or the group that received GTS-21 alone. Notably, mice treated with GTS-21 prior to HDM exposure exhibited significantly lower inflammatory cell infiltration in these regions than those treated with PBS (**Figures 2C, D**). Therefore, GTS-21 effectively attenuated airway inflammation in this murine model of allergic airway inflammation.

### 3.2 Pretreatment with GTS-21 attenuates TSLP and type 2-associated cytokine secretion in response to HDM in lung tissue

**Figure 3:**
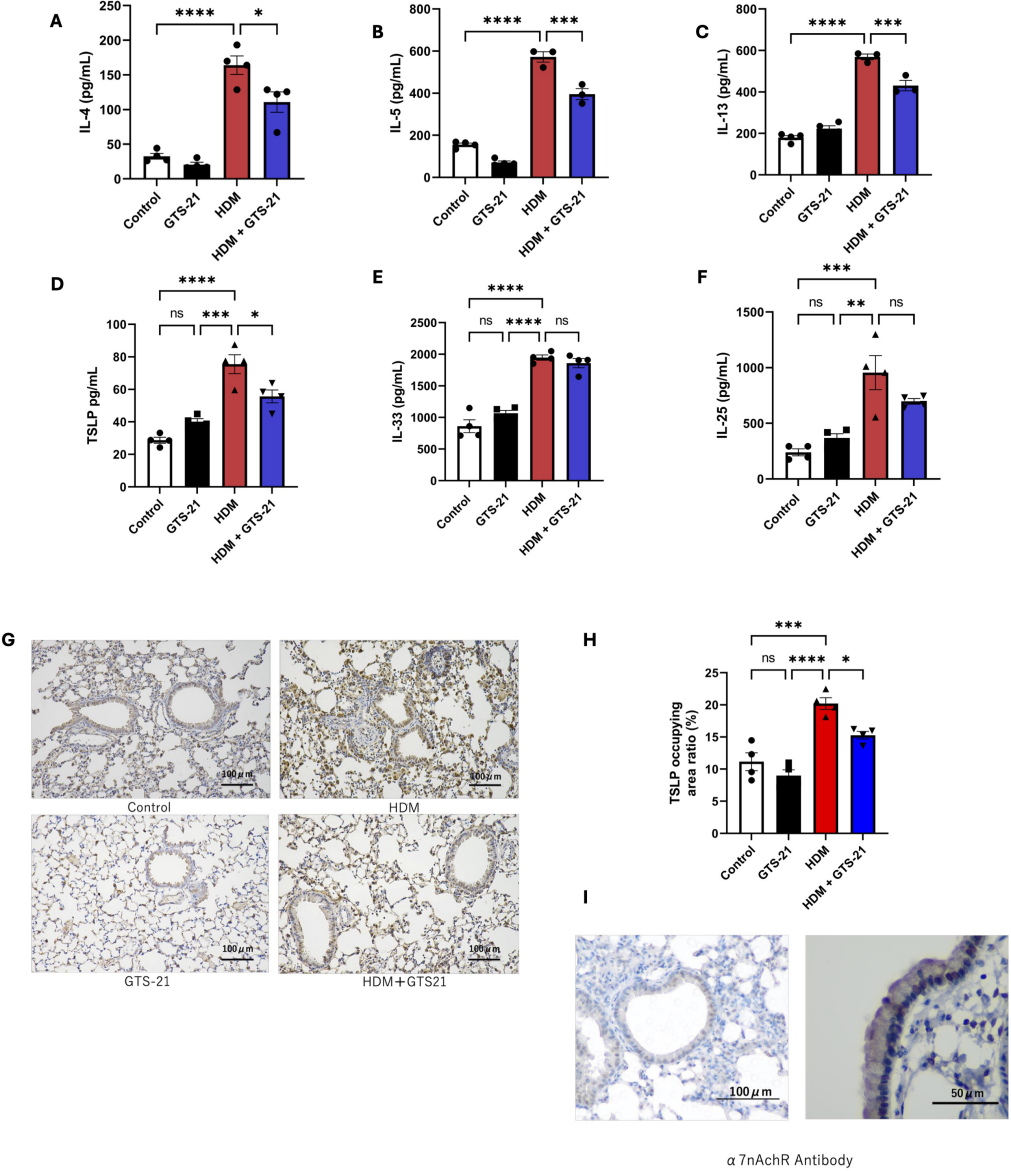
GTS-21 suppresses TSLP and type 2-related cytokines in the lungs of a murine model of HDM-induced allergic airway inflammation **(A–F)** Levels of type 2-related cytokines (IL-4, IL-5, and IL-13) and epithelial cell-derived cytokines (TSLP, IL-33, and IL-25) were measured following intratracheal administration of HDM (30 μg) or saline and GTS-21 (125 μg) or PBS to female C57BL/6J mice. Cytokine concentrations in lung homogenate supernatants were analyzed: **(A)** IL-4, **(B)** IL-5, **(C)** IL-13, **(D)** TSLP, **(E)** IL-33, and **(F)** IL-25 (n = 3–4). **(G)** Immunohistochemical staining of TSLP in lung sections. Scale bar = 100 μm; n = 4 mice. **(H)** Quantification of TSLP-positive area as a percentage of the total area using ImageJ software. Data are representative of at least two independent experiments and are presented as means ± standard error of the mean. Statistical analysis was performed using two-way analysis of variance. ∗P < 0.05, ∗∗P < 0.01, ∗∗∗P < 0.001, ∗∗∗∗P < 0.0001. **(I)** Immunohistochemistry for α7nAChR in lung sections from untreated wild-type mice, showing positive cytoplasmic staining in airway epithelial cells. TSLP, thymic stromal lymphopoietin; HDM, house dust mite; IL, interleukin; PBS, phosphate-buffered saline; α7nAChR, alpha7 nicotinic acetylcholine receptor.

Lung tissues were harvested from the mice described above, and cytokine concentrations in the lung homogenate supernatants were quantified using ELISA. In mice intratracheally exposed to HDM, levels of type 2-related cytokines (IL-4, IL-5, and IL-13) and TSLP were significantly higher than those in the control group. However, in mice pretreated with GTS-21, the levels of these cytokines were significantly lower than those in the PBS-treated group (**Figures 3A–F**).

Immunohistochemical analysis of lung sections revealed markedly increased TSLP expression in the HDM-exposed group, whereas TSLP-positive areas were significantly reduced in the GTS-21-treated group (**Figures 3G, H**). Additionally, immunohistochemistry using lung tissue from untreated wild-type mice demonstrated that α7nAChR is expressed in airway epithelial cells (**Figure 3I**). These findings suggest that GTS-21 treatment is associated with reduced TSLP expression in the airways and decreased downstream type 2 cytokine production *in vivo*. Together with the *in vitro* findings, these results are consistent with the possibility that airway epithelial responses contribute to the observed anti-inflammatory effects.

### 3.3 GTS-21 reduces TSLP secretion and is associated with increased HO-1 expression in BEAS-2B airway epithelial cells under LPS and HDM stimulation

**Figure 4:**
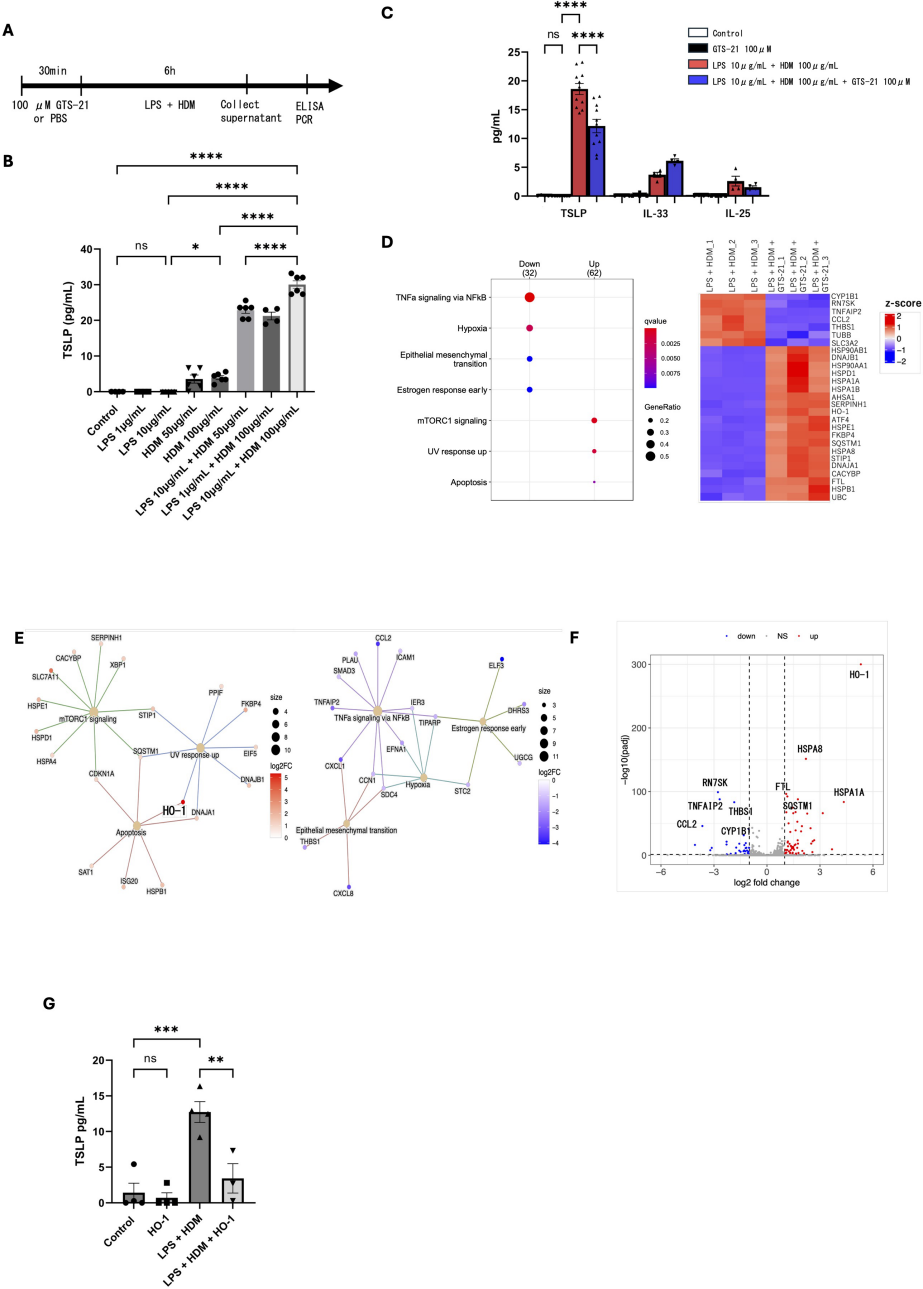
GTS-21 reduces TSLP secretion and is associated with increased HO-1 expression in BEAS-2B airway epithelial cells under LPS and HDM stimulation **(A)** Assessment of TSLP secretion in BEAS-2B cells following stimulation with LPS and HDM and the inhibitory effect of GTS-21. Cells were pretreated with GTS-21 (100 μM) for 30 min, followed by HDM (100 μg/mL) and LPS (10 μg/mL) exposure for 6 h. **(B)** ELISA analysis of TSLP levels in culture supernatants after 6-h incubation with LPS (1 or 10 μg/mL) or with LPS and HDM (50 or 100 μg/mL) (n = 4–6). Under these conditions, combined LPS and HDM stimulation induced TSLP more reproducibly than HDM alone. **(C)** ELISA analysis of TSLP, IL-33, and IL-25 levels in cell supernatants from BEAS-2B cells treated under similar conditions (n = 4–11). In panels B and C, BEAS-2B cells were stimulated with LPS (10 μg/mL) and HDM (100 μg/mL) for 6 h. **(D)** RNA-seq results from BEAS-2B cells stimulated with LPS and HDM, with or without GTS-21 treatment (n = 3). A total of 15,472 genes were identified. Differential expression analysis revealed 32 downregulated genes and 62 upregulated genes in GTS-21-treated cells compared to untreated cells (false discovery rate < 0.05). **(E)** Enrichment analysis of differentially expressed genes. **(F)** Volcano plot illustrating gene expression changes in response to GTS-21. **(G)** ELISA analysis of TSLP levels in cell supernatants from BEAS-2B cells pretreated with recombinant HO-1 (1 ng/mL) for 30 min, followed by HDM (100 μg/mL) and LPS (10 μg/mL) exposure for 6 h (n = 3–4). Data are representative of at least two independent experiments and are represented as means ± standard error of the mean. Statistical analysis in panels B and C was performed using one-way analysis of variance; analysis in panel D was performed using Student’s t-test. ∗P < 0.05, ∗∗P < 0.01, ∗∗∗P < 0.001, ∗∗∗∗P < 0.0001. TSLP, thymic stromal lymphopoietin; HDM, house dust mite; α7nAChR, alpha7 nicotinic acetylcholine receptor; LPS, lipopolysaccharide; ELISA, enzyme-linked immunosorbent assay; HO-1, heme oxygenase-1.

**Figure 5:**
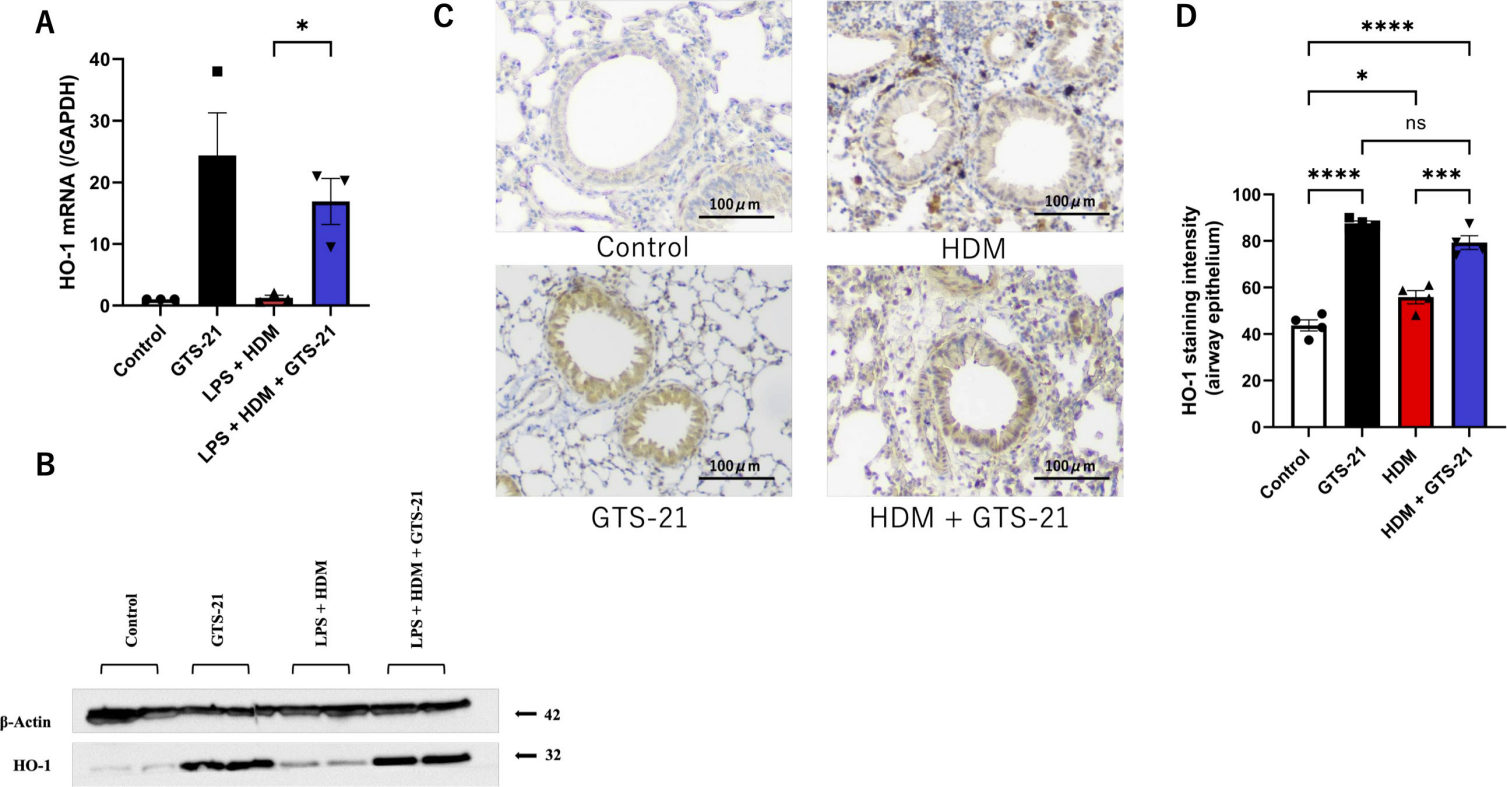
qPCR, western blot, and immunohistochemical analyses show increased HO-1 expression following HDM or GTS-21 treatment **(A)** HMOX1 (HO-1) mRNA expression in BEAS-2B cells measured using qPCR (n = 3). **(B)** HO-1 and β-actin protein expression levels assessed by western blotting. **(C)** HO-1 immunohistochemistry of lung sections. **(D)** Quantification of HO-1 immunostaining. Data are representative of at least two independent experiments and are represented as means ± standard error of the mean. Statistical analysis in panel A was performed using one-way analysis of variance. ∗P < 0.05, ∗∗P < 0.01, ∗∗∗P < 0.001, ∗∗∗∗P < 0.0001. HO-1, heme oxygenase-1; qPCR, quantitative real-time PCR Supplementary. Flow cytometry gating strategy. Single-cell suspensions were first gated based on forward scatter (FSC) and side scatter (SSC) to exclude debris, followed by doublet discrimination using FSC-A and FSC-H parameters. Dead cells were excluded using a viability dye. Leukocytes were then identified as CD45^+^ cells, and subsequent gating was performed to identify specific immune cell populations based on the expression of lineage and surface markers. And group 2 innate lymphoid cells (ILC2) were defined as CD45^+^Lin^−^CD127^+^ST2^+^ cells.

Based on the findings above, we hypothesized that airway epithelial responses may contribute to the anti-inflammatory effects associated with GTS-21 treatment *in vivo*. To further investigate this possibility, we assessed the effect of GTS-21 on TSLP secretion in human airway epithelial BEAS-2B cells. As low-level endotoxin/TLR4 signaling can enhance allergic responses (23–26), BEAS-2B cells were stimulated with 10 μg/mL LPS and either 100 μg/mL HDM for 6 h to establish a reproducible TSLP induction system. TSLP levels in the supernatant were measured using ELISA (**Figure 4A**). LPS and HDM co-stimulation resulted in significantly higher TSLP secretion than that observed with LPS alone or in the unstimulated control (**Figure 4B**). Under our experimental conditions, combined LPS and HDM stimulation induced TSLP more reproducibly than HDM alone. To evaluate the effect of α7nAChR activation, BEAS-2B cells were pretreated with 100 μM GTS-21 or PBS for 30 min, followed by LPS and HDM stimulation for 6 h. TSLP production was significantly lower in the GTS-21-treated group than in the PBS-treated group. IL-33 and IL-25 concentrations remained very low and were near or below the lower detection range in most samples (**Figure 4C**). To elucidate the mechanism underlying TSLP suppression, RNA-seq analysis was performed on RNA extracted from BEAS-2B cells treated with GTS-21 or PBS under the same stimulation conditions. We identified 15,472 genes and performed a pairwise comparison of expression between the two groups. GTS-21-treated cells exhibited greater downregulation of 32 genes and upregulation of 62 genes than those in PBS-treated cells (false discovery rate < 0.05) (**Figure 4D**). Enrichment analysis revealed significant upregulation of genes associated with cellular protection via mTORC1 signaling and apoptosis regulation. Additionally, genes related to tumor necrosis factor-α signaling via nuclear factor kappa-light-chain enhancer of activated B cells (NF-κB) were downregulated, indicating that GTS-21 stimulation may suppress inflammatory cytokine pathways while promoting cell-protective responses (**Figure 4E**). In the volcano plot analysis, HMOX1 (HO-1) was one of the most significantly upregulated genes in GTS-21-treated cells (**Figure 4F**). Instead of GTS-21, pretreatment with 1 ng/mL human HO-1 protein for 30 min, followed by LPS and HDM stimulation, resulted in significantly reduced TSLP production in the HO-1 protein-treated group. HO-1 induction alone did not significantly affect TSLP levels compared with the control group (**Figure 4G**). qPCR and western blotting confirmed a significant increase in HO-1 expression at both the mRNA and protein levels in BEAS-2B cells following GTS-21 stimulation, regardless of LPS and HDM exposure (**Figures 5A, B**). Finally, immunohistochemical analysis of lung sections (**Figures 5C, D**) revealed increased HO-1 staining in the airway epithelium of GTS-21-treated mice. Together, these findings indicate that GTS-21 treatment is associated with increased HO-1 expression and reduced TSLP production under the present experimental conditions.

## 4 Discussion

In this study, we found that intratracheal administration of GTS-21, an α7nAChR partial agonist, significantly attenuated airway inflammatory responses in a murine model of HDM-induced allergic airway inflammation. GTS-21 exhibits partial agonism at α7nAChR and has relative (not absolute) selectivity across nAChR subtypes, depending on experimental conditions and concentration (27–29). GTS-21 treatment markedly reduced eosinophilic infiltration and suppressed the expression of type 2 cytokines, including IL-5 and IL-13, as well as the epithelial-derived alarmin TSLP. *In vitro* experiments using BEAS-2B airway epithelial cells further showed that GTS-21 reduced TSLP secretion in response to LPS and HDM stimulation, supporting the possibility that airway epithelial responses contribute to the observed anti-inflammatory effects. RNA-seq revealed that GTS-21 upregulates HO-1 expression while downregulating the activation of NF-κB, implicating a potential intracellular pathway associated with its anti-inflammatory effects.

These findings align with previous reports indicating CAP activation suppresses allergic airway inflammation (4, 30). Our results support a potential role for cholinergic receptor-associated signaling in the regulation of epithelial TSLP production during allergic airway inflammation. Given the pivotal role of TSLP in initiating and amplifying allergic responses and promoting corticosteroid resistance in ILC2s, modulation of epithelial cytokine responses through this pathway may represent a potential area for further therapeutic investigation (12, 31).

HO-1 induction may contribute to the anti-inflammatory effects associated with GTS-21 treatment. HO-1 is a stress-inducible, cytoprotective enzyme with antioxidant, anti-apoptotic, and anti-inflammatory effects (32). Lv et al. (33) demonstrated that HO-1 suppresses allergic airway inflammation by inhibiting pyroptosis in airway epithelial cells. Mechanistically, HO-1 binds to the Rel homology domain of NF-κB p65, thereby disrupting NF-κB-dependent pyroptotic signaling. This interaction interferes with gasdermin D (GSDMD)-mediated pore formation and limits TSLP release. Furthermore, pharmacological induction of HO-1 using hemin resulted in reduced epithelial pyroptosis, lower TSLP levels, and attenuated type 2 inflammation in an HDM-induced allergic airway inflammation model. Notably, administration of disulfiram—a GSDMD pore formation inhibitor—produced comparable anti-inflammatory effects (33). In our study, GTS-21 treatment was associated with increased HO-1 expression in BEAS-2B cells and in airway epithelium, while recombinant HO-1 reduced TSLP production under LPS and HDM stimulation. Together, these findings support HO-1 as a candidate mediator, although a causal requirement for HO-1 was not established in the present study.

While previous studies have primarily focused on the immunomodulatory effects of α7nAChR agonists on immune cells, such as macrophages and ILC2s (34, 35), our findings suggest that the airway epithelium may be one important site of cholinergic regulation in allergic airway inflammation. However, because intratracheal GTS-21 administration may affect multiple α7nAChR-expressing cell populations *in vivo*, the present data do not establish an exclusively epithelial-intrinsic mechanism. Accordingly, the potential utility of inhaled α7nAChR-targeting agents should be regarded as a future direction requiring additional investigation.

This study has several limitations. First, our *in vivo* findings are based on a single murine model of allergic airway inflammation and did not include assessment of airway hyperresponsiveness or lung function. Second, although GTS-21 treatment was associated with HO-1 upregulation, HO-1 loss-of-function experiments were not performed; therefore, a causal role for HO-1 cannot be concluded. Third, because GTS-21 has relative rather than absolute selectivity and we did not include pharmacological antagonism or genetic loss-of-function approaches, receptor specificity remains to be confirmed. Fourth, because only female mice were used, potential sex-dependent differences require future investigation. Fifth, the BEAS-2B model used LPS with HDM to establish reproducible TSLP induction, which may not fully reflect physiological allergen exposure.

In conclusion, GTS-21 attenuated allergic airway inflammation and suppressed epithelial TSLP production in association with increased HO-1 expression. These findings suggest that cholinergic receptor-associated signaling may modulate airway epithelial inflammatory responses and warrant further investigation using receptor-specific and loss-of-function approaches.

## Data Availability

The data presented in the study are deposited in the NCBI Sequence Read Archive (SRA) repository under BioProject accession number PRJNA1449248. The dataset is publicly available at: https://www.ncbi.nlm.nih.gov/sra/PRJNA1449248.

## Supplementary material

Supplementary Figure 1 Flow cytometry gating strategy.
