# Supplementary figures and images for: Alpha7 nicotinic acetylcholine receptor activation attenuates allergic airway inflammation and is associated with heme oxygenase-1 induction
Source: Front Immunol. 2026 Apr 23;17:1808271. doi: 10.3389/fimmu.2026.1808271 (PMC13149419; doi:10.3389/fimmu.2026.1808271)

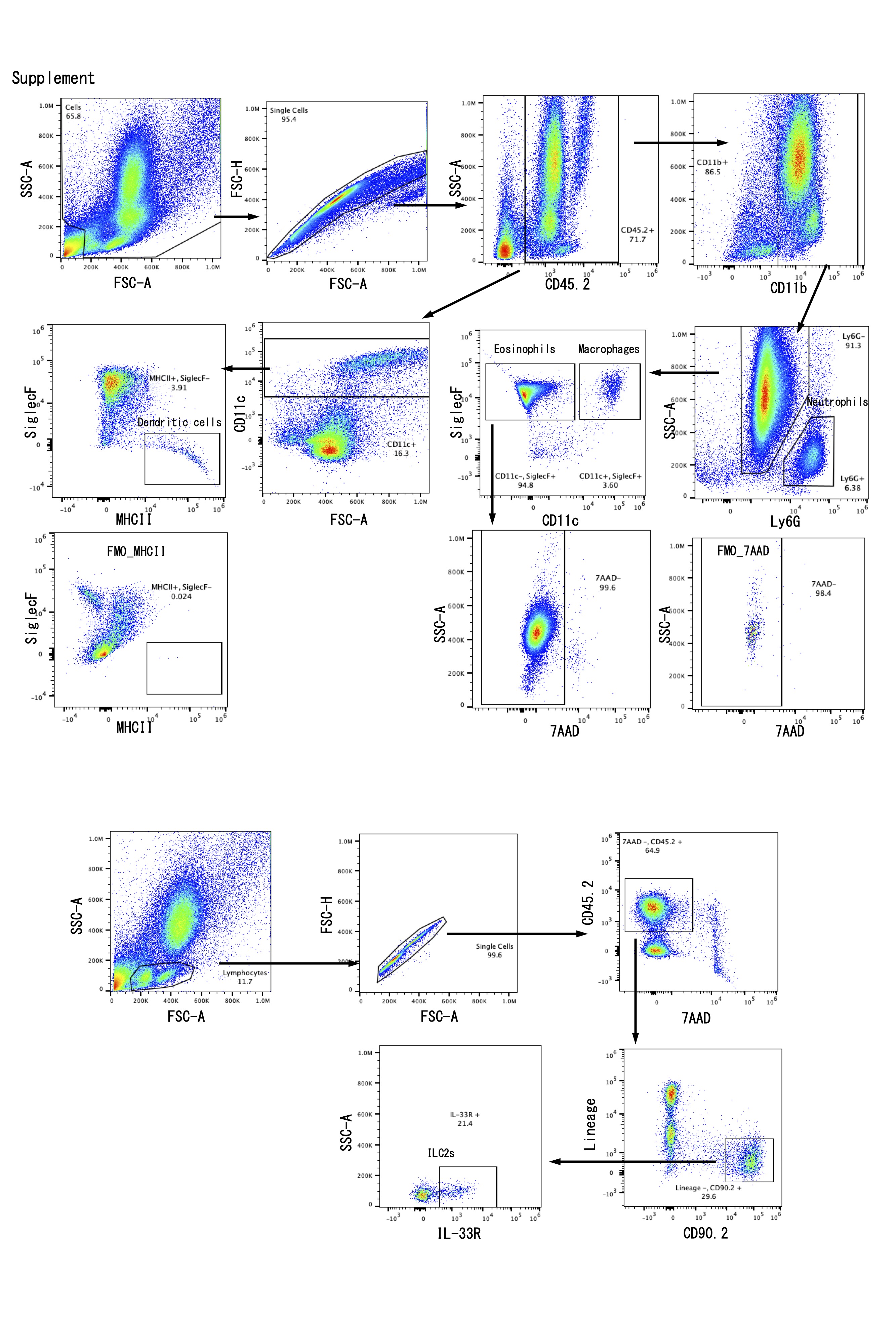

Supplement: Supplementary Figure 1 — Flow cytometry gating strategy. [file Image1.jpeg]
